# Dissecting the contribution of O-Antigen and proteins to the immunogenicity of *Shigella sonnei* generalized modules for membrane antigens (GMMA)

**DOI:** 10.1038/s41598-020-80421-y

**Published:** 2021-01-13

**Authors:** Francesca Mancini, Gianmarco Gasperini, Omar Rossi, Maria Grazia Aruta, Maria Michelina Raso, Renzo Alfini, Massimiliano Biagini, Francesca Necchi, Francesca Micoli

**Affiliations:** 1 GSK Vaccines Institute for Global Health (GVGH), via Fiorentina 1, 53100 Siena, Italy; 2 GSK, Siena, Italy

**Keywords:** Carbohydrates, Biochemistry, Diseases, Infectious diseases, Immunological techniques, Isolation, separation and purification, Mass spectrometry, Molecular engineering, Proteomic analysis

## Abstract

GMMA are exosomes released from engineered Gram-negative bacteria resembling the composition of outer membranes. We applied the GMMA technology for the development of an O-Antigen (OAg) based vaccine against *Shigella sonnei*, the most epidemiologically relevant cause of shigellosis. *S. sonnei* OAg has been identified as a key antigen for protective immunity, and GMMA are able to induce anti-OAg-specific IgG response in animal models and healthy adults. The contribution of protein-specific antibodies induced upon vaccination with GMMA has never been fully elucidated. Anti-protein antibodies are induced in mice upon immunization with either OAg-negative and OAg-positive GMMA. Here we demonstrated that OAg chains shield the bacteria from anti-protein antibody binding and therefore anti-OAg antibodies were the main drivers of bactericidal activity against OAg-positive bacteria. Interestingly, antibodies that are not targeting the OAg are functional against OAg-negative bacteria. The immunodominant protein antigens were identified by proteomic analysis. Our study confirms a critical role of the OAg on the immune response induced by *S. sonnei* GMMA. However, little is known about OAg length and density regulation during infection and, therefore, protein exposure. Hence, the presence of protein antigens on *S. sonnei* GMMA represents an added value for GMMA vaccines compared to other OAg-based formulations.

## Introduction

Several bacteria are cause of devastating burden of disease in low- and middle-income countries (LMICs), for which vaccines are not available due to a lack of need in developed countries and consequent absence of financial reward derived from sales. One of such pathogens is *Shigella sonnei*, the most epidemiologically relevant causative agent of human shigellosis^1,2^. According to the Global Burden of Disease estimates, *Shigella* is the second most deadly diarrheal disease, causing approximately 212,438 deaths per year, globally^3^. Of these, 63,713 deaths are in children under five years of age, the majority of which are in LMICs.

Due to increasing resistance to treatment with antibiotics, *Shigella* is in the World Health Organization (WHO) Anti Microbial Resistance (AMR) priority list^4^. Moreover, *Shigella* vaccines are considered a priority for LMICs by WHO’s Product Development for Vaccines Advisory Committee (PDVAC) with the aim to reduce diarrhea, dysentery and morbidity in children under 5 years of age^5^. Thus, a safe and affordable *Shigella* vaccine to be administered to infants is an important public health priority.

A first-generation glycoconjugate vaccine composed of *S. sonnei* O-Antigen (OAg) linked to *Pseudomonas aeruginosa* recombinant exoprotein A (*r*EPA) was developed over 20 years ago and elicited protective antibodies against surface OAg in young adults^6^. Antibody titers, however, decreased with the age together with observed protection. Indeed, while efficacy of 71% was observed in 3–4 years olds, it dropped to zero in the age group of infants and toddlers^7^. Other *Shigella* OAg-based vaccines, making use of different manufacturing platforms, are currently in clinical development and have demonstrated their immunogenicity^8–11^.

Generalized Modules for Membrane Antigens (GMMA) are exosomes released from Gram-negative bacteria genetically engineered to enhance their production through the disruption of the bacterial envelope integrity and to minimize their capacity to promote reactogenic responses once injected, e.g. through modification of the lipid A moiety of the lipopolysaccharide (LPS)^12,13^. GMMA resemble the composition of the bacterial outer membrane, presenting to the immune system LPS and outer membrane proteins in their native environment. GMMA are self-adjuvanting through delivery of innate signals like pathogen-associated molecular patterns (PAMPs) and favour the uptake by antigen-presenting cells due to their optimal size. The potential for a strong immune response is associated to the simplicity of manufacturing, that makes GMMA an attractive platform especially for the development of vaccines for LMICs^14–16^.

We have developed a GMMA-based vaccine to prevent *S. sonnei* infections using a *S. sonnei* strain that was genetically modified to produce high yield particles with penta-acylated lipid A, with reduced endotoxicity and to stably maintain the virulence plasmid encoding for the immunodominant OAg component of the LPS^14^. *S. sonnei* GMMA showed to be well tolerated and to elicit anti-LPS antibodies in clinical trials in healthy european adults and endemic kenyan population^17–19^. This is part of wider strategy to obtain a multivalent OAg-GMMA vaccine covering *S. sonnei* and the most prevalent *S. flexneri* serotypes and protecting endemic populations from pediatric deadly diarrhea.

*S. Sonnei* GMMA display on their surface not only the OAg but also *Shigella* surface proteins and periplasmic proteins are entrapped in their lumen as well. Even though GMMA can be employed as a vehicle to deliver *Shigella* OAg to the immune system, GMMA proteins can also be potent immunogens. However, the quality and the contribution to the overall immune response of the GMMA protein-specific antibodies have never been elucidated in full, especially when the OAg is present^15^. Therefore, in this study we aimed at investigating proteins contribution to the immune response induced by *S. sonnei* GMMA, both in presence and absence of the key OAg target.

We confirmed that anti-proteins antibodies are elicited in mice after immunization with both OAg-positive and OAg-negative GMMA, but only LPS-specific antibodies (produced after immunization with OAg-positive GMMA) can bind to OAg-positive *S. sonnei* bacterial surface and determine complement mediated killing. However, when bacteria are not shielded by OAg chains, antibodies specific to GMMA proteins can bind to the bacterial surface as well and this results in the killing of the pathogen.

## Results

### GMMA production and characterization

GMMA displaying the OAg on their surface and carrying wild type lipid A were produced from a *S. sonnei* 53G strain (Ss OAg+) (Table 1). Further mutations were introduced in this GMMA producer strain to decrease the number of the lipid A acyl chains, thus obtaining two alternative strains carrying a lipid A with reduced endotoxicity: Ss Δ*htrB* OAg+ and Ss Δ*msbB* OAg+ (Table 1). GMMA not displaying the OAg on their surface were produced from a *S. sonnei* 53G strain cured from the big virulence plasmid (Ss OAg−). The same lipid A modifications were also applied to the OAg-negative strain to obtained detoxified GMMA (Ss *ΔhtrB* OAg− and Ss *ΔmsbB* OAg−) (Table 1).

**Table 1:** Strains used in the study.

Abbreviation	Genotype	Description	Reference
Ss OAg−	*S. sonnei*-pSS *tolR*::*kan*	Hyperblebbing *S. sonnei*-pSS. LPS OAg-deficient rough mutant with hexa-acylated lipid A	^15^
Ss Δ*msbB* OAg−	*S. sonnei*-pSS *tolR*::*kan msbB1::cat*	Hyperblebbing *S. sonnei*-pSS. LPS OAg-deficient rough mutant with penta-acylated (-myristoyl chain) lipid A	^12^
Ss Δ*htrB* OAg−	*S. sonnei* -pSS *tolR*::*kan htrB::cat*	Hyperblebbing *S. sonnei*-pSS. LPS OAg-deficient rough mutant with penta-acylated (-lauroyl chain) lipid A	^12^
Ss OAg+	*S. sonnei virG::nadAB tolR*::*kan*	Hyperblebbing *S. sonnei*. pSS stabilized, OAg positive with hexa-acylated lipid A	^14^
Ss Δ*msbB* OAg+	*S. sonnei tolR*::*kan virG::nadAB msbB1::cat msbB2::ery*	Hyperblebbing *S. sonnei*. pSS stabilized, OAg-positive with penta-acylated (-myristoyl chain) lipid A	This study
Ss Δ*htrB* OAg+	*S. sonnei virG::nadAB tolR*::*kan htrB::cat*	Hyperblebbing *S. sonnei*. pSS stabilized, OAg-positive with penta-acylated (-lauroyl chain) lipid A	^14^
Ss WT OAg+	*S. sonnei virG::cat*	Growth in the presence of chloramphenicol selects for the presence of pSS and thus avoids loss of the OAg cluster in vitro	^20^
Ss WT OAg−	*S. sonnei wbg::cat*	LPS OAg-deficient rough mutant. Knock-out of the OAg biosynthesis cluster (genes *wzz* to *wbgZ*)	^20^

The table summarizes all the bacterial strains used in this study.

**Figure 1:**
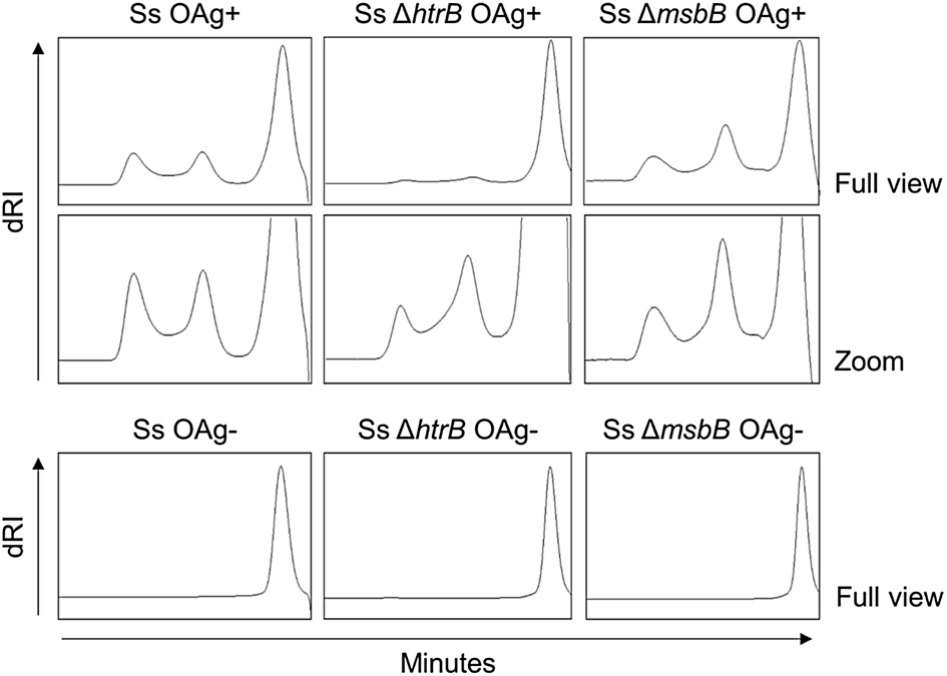
HPLC-SEC profiles of sugars extracted from *S. sonnei* GMMA. Detection by refractive index of different OAg populations. Results of one representative experiment performed in technical duplicates.

All GMMA were fully characterized and showed an average radius in the range of 30–50 nm as measured by High Performance Liquid Chromatography-Size Exclusion Chromatography (HPLC-SEC) coupled with Multiangle Light Scattering (MALS) (Table 2). Absence of free soluble proteins or DNA fragments was confirmed by HPLC-SEC analysis performed on the entire particles (Fig. S1a). The expected lipid A structure was confirmed by Matrix-Assisted Laser Desorption/Ionization-Time Of Flight Mass Spectrometry (MALDI-TOF MS) analysis and the ability of the different GMMA to activate Toll-like receptor 4 (TLR-4) was verified in a Monocyte Activation Test (MAT) (Fig. S1b,c). OAg-positive GMMA displayed a low molecular weight (LMW) OAg population, a medium molecular weight (MMW) OAg population and a high molecular weight group 4 capsule (G4C)^20^ with average size of approximately 2.0 kDa, 20 kDa and 200 kDa respectively (Fig. 1). All polysaccharide species shared the expected structure, consisting of a disaccharide repeat containing 2-acetamido-2-deoxy-l-altruronic acid (l-AltNAcA) and 2-acetamido-2-deoxy-l-fucose (FucNAc4N), as verified by Nuclear Magnetic Resonance (^1^H NMR) analysis. However, GMMA from Ss *ΔhtrB* OAg+ differed from the other two GMMA samples in terms of OAg to protein weight ratio. In particular, the *htrB* mutation had a strong impact on the overall OAg density which was around 8 times lower compared to the parental strain and the alternative *msbB* double mutant (Table 2).

**Table 2:** Analytical characterization of the antigens.

GMMA	Lipid A type (MALDI-TOF)	Radius (nm) (HPLC-SEC/MALS)	OAg/protein w/w ratio (HPAEC-PAD/micro BCA)
Ss OAg+	Hexa	35.0	0.25
Ss Δ*htrB* OAg+	Penta	47.0	0.03
Ss Δ*msbB* OAg+	Penta	42.0	0.22
Ss OAg−	Hexa	41.5	No OAg detected
Ss Δ*htrB* OAg−	Penta	29.3	No OAg detected
Ss Δ*msbB* OAg−	Penta	33.7	No OAg detected

The table summarizes GMMA characterization in terms of lipid A structure (MALDI-TOF), size (HPLC-SEC/MALS) and OAg to protein (w/w) ratio (HPAEC-PAD/micro BCA).

For the OAg-negative GMMA, the absence of OAg repeats was confirmed by High-Performance Anion-Exchange Chromatography coupled with Pulsed Electrochemical Detection (HPAEC-PAD) analysis, with lack of the characteristic sugars of the OAg repeating unit. Also, HPLC-SEC analysis on extracted sugars showed absence of G4C and MMW OAg and only presence of core molecules at 1.8 kDa (Fig. 1).

### Proteomic analysis of *S. sonnei* GMMA

**Figure 2:**
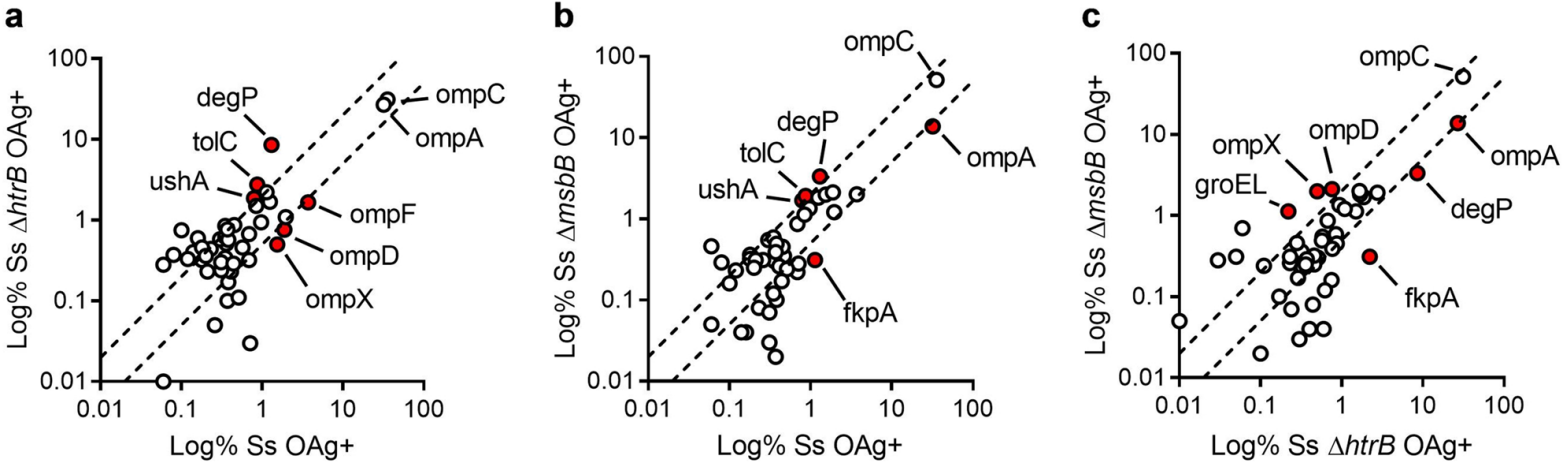
Proteomic analysis of *S. sonnei* GMMA (most abundant proteins). Proteomic analysis was performed by LC–MS/MS. A global set of 150 proteins were quantified in the six different samples. The abundance of each protein in the OAg-positive GMMA with hexa-acylated lipid A was compared with that of the two OAg-positive GMMA with penta-acylated lipid A (**a**,**b**). Moreover, the abundance of each protein in the two OAg-positive GMMA with penta-acylated lipid A was compared. Results represent one experiment performed in technical triplicates.

Proteomic analysis was performed by Liquid Chromatography coupled with Mass Spectrometry (LC–MS/MS). A global set of 250 proteins were quantified analyzing the six different GMMA samples (Table S1). We decided to focus on the 30 most abundant proteins in each GMMA preparation, which accounted for > 95% of the total protein composition of each sample. We obtained a list of 49 proteins in total for the six samples (Table S2). The localization of the quantified proteins was predicted according to PSORTb software. The analysis predicted ~ 90% of the total protein amount for each GMMA sample to be either outer membrane proteins or periplasmic proteins (Table S2), thus confirming that GMMA are generated by outer membrane blebbing and not by bacterial lysis. Around 2–5% of the total protein amount had an unknown prediction of localization in all samples but only a minority of proteins was predicted to be inner membrane or cytoplasmic. Next, we compared the abundance of each protein in the different GMMA samples (Table S2 and Fig. 2). In particular, we compared the OAg+ GMMA with penta-acylated lipid A to each other and to the OAg+ GMMA with hexa-acylated lipid A (Fig. 2). The results of the comparisons are reported as XY plots where proteins showing similar abundances in the two compared samples lay between the two dotted lines, while proteins showing at least a twofold difference in one of the two samples lay outside the dotted lines. Only proteins accounting for at least 1% of the total composition and varying of at least twofold in the compared samples were considered significantly different and were depicted as red circles. The different comparisons showed that the majority of proteins are similarly represented in the different GMMA samples, while only 10 proteins are slightly variable. In particular the most evident changes, due to the two modifications of the lipid A structure, can be highlighted in a decrease on protein abundance (i.e. OmpA, OmpD, OmpX, OmpF, FkpA) or an increase (i.e. DegP, UshA, TolC).

### Immunogenicity studies in mice

OAg-positive and OAg-negative GMMA with penta-acylated lipid A were compared in mice at the same dose of GMMA proteins of 10 µg to maximise the immune response against GMMA proteins. As a consequence of the different OAg density in the OAg-positive GMMA (Table 2), the group of mice immunized with Ss Δ*msbB* OAg+ GMMA received a dose of OAg which was around 8 times higher compared to the group of mice immunized with Ss Δ*htrB* OAg+ GMMA.

#### Serological response

**Figure 3:**
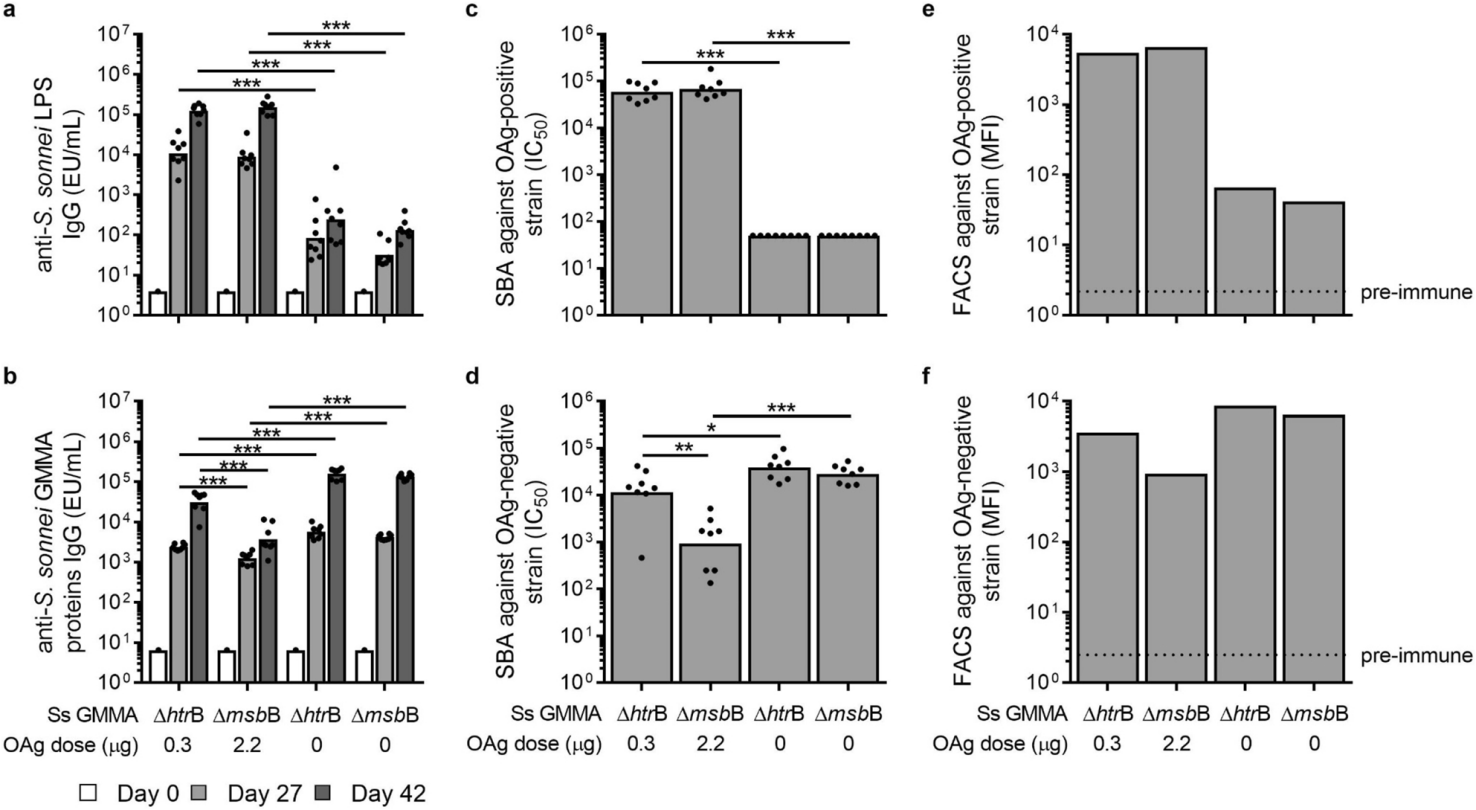
Immunogenicity study of GMMA in mice: analysis of serum IgG content and functionality. Single sera were collected from the differently immunized groups (8 mice/group). Single sera have been analysed in ELISA using as coating antigens *S. sonnei* full LPS (**a**) or OAg-negative GMMA (**b**) and in SBA against OAg-positive (**c**) and OAg-negative (**d**) *S. sonnei* strains. Geometric means and individual values are plotted in the graphs. Mann–Whitney test was performed between the two groups immunized with OAg-positive GMMA, the two groups immunized with OAg-negative GMMA and between the groups immunized with GMMA sharing the same lipid A structure (*P < 0.05, **P < 0.01, ***P < 0.001). *S. sonnei* bacteria displaying (**e**) or not (**f**) the OAg have been stained with pooled sera from the study and analyzed by FACS. Mean Fluorescence Intensity (MFI) of 10,000 aquired events is reported.

Independently from the different OAg dose, Ss Δ*htrB* OAg+ GMMA and Ss Δ*msbB* OAg+ GMMA elicited comparable levels of anti-LPS specific total IgG both 27 days after the first immunization and 14 days after the second one, with titers at day 42 being statistically higher than those at day 27. Also OAg-negative GMMA elicited anti-LPS IgG titers, even if significantly lower than those elicited by vaccination with the corresponding OAg-positive GMMA. Anti-LPS titers observed after vaccination with OAg-negative GMMA were reasonably due to the presence of anti-LPS core antibodies (considering that *S. sonnei* LPS was used as coating antigen in ELISA) and were similar in the two groups of mice immunized with GMMA lacking the OAg (Fig. 3a).

Anti-LPS specific IgM resulted to be lower for mice immunized with Ss Δ*htrB* OAg+ GMMA compared to Ss Δ*msbB* OAg+ GMMA, whereas titers were comparable between the two groups of mice immunized with OAg-negative GMMA. Again, anti-LPS specific IgM were higher after vaccination with OAg-positive GMMA than with OAg-negative GMMA (Fig. S2a). All anti-LPS specific IgG subclasses (IgG1, IgG2a, IgG2b, and IgG3) were elicited upon immunization with both OAg-positive and OAg-negative GMMA (Fig. S3). LPS-specific IgG subclasses were comparable among the two groups immunized with OAg-positive GMMA and the two groups of mice immunized with OAg-negative GMMA respectively, both at study day 27 and 42. Interestingly, only the groups immunized with OAg-negative GMMA were characterised by IgG1/IgG2 ratios < 1 (Fig. S2c).

Protein-specific total IgG elicited by vaccination with OAg-negative GMMA were significantly higher than those elicited by vaccination with the corresponding OAg-positive GMMA both after the first and the second immunization. Moreover, IgG elicited by Ss Δ*htrB* OAg+ GMMA were higher than those induced by immunization with Ss Δ*msbB* OAg+ GMMA (Fig. 3b).

Also anti-protein specific IgM were higher for the group immunized with Ss Δ*htrB* OAg+ GMMA compared to Ss Δ*msbB* OAg+ GMMA, whereas antibody levels were comparable between the two groups of mice immunized with OAg-negative GMMA. Anti-GMMA proteins specific IgM were lower after vaccination with OAg-positive GMMA than with corresponding OAg-negative GMMA (Fig. S2b). As for anti-LPS specific IgG subclasses, all anti-GMMA proteins specific IgG subclasses (IgG1, IgG2a, IgG2b, and IgG3) were elicited upon immunization with both OAg-positive and OAg-negative GMMA (Fig. S3). IgG subclasses were comparable among the two groups immunized with OAg-negative GMMA, both at study day 27 and 42, whereas anti-GMMA proteins specific IgG subclasses were higher after immunization with Ss Δ*htrB* OAg+ GMMA compared to Ss Δ*msbB* OAg+ GMMA. IgG1/IgG2 ratios were < 1 for all the groups. IgG1/IgG2 ratio obtained with Ss Δ*htrB* OAg+ GMMA was statistically different from that of Ss Δ*msbB* OAg+ GMMA, the latter being lower (Fig. S2d).

#### Vaccine-elicited antibody functionality

Functionality of the sera was evaluated against an OAg-positive *S. sonnei* strain through a serum bactericidal assay (SBA) based on luminescence (Fig. 3c)^21^. Similar killing of *S. sonnei* was observed after immunization with OAg-positive GMMA and no killing after immunization with OAg-negative GMMA. We wondered if this could be due to the fact that anti-proteins antibodies could not bind to the bacterial surface proteins shielded by the OAg chains. Therefore, we also evaluated bactericidal activity of sera against an OAg-negative *S. sonnei* strain (Fig. 3d). SBA titers for sera raised against OAg-negative GMMA were higher compared to those raised against OAg-positive GMMA. Sera raised against Ss Δ*msbB* OAg+ GMMA showed lower SBA titers compared to sera raised against Ss Δ*htrB* OAg+ GMMA, similarly to what observed in terms of anti-proteins total IgG levels (Fig. 3b).

#### Vaccine-elicited antibody binding to bacteria

To further confirm that anti-proteins antibodies were not able to bind to the surface of bacteria due to the presence of the OAg chains, the same bacteria used for SBA were stained with pooled sera representing the four immunization groups and analyzed by Fluorescence Activated Cell Sorting (FACS). When OAg-positive bacteria were stained with sera from mice immunized with OAg-positive GMMA, the observed Mean Fluorescence Intensities (MFI) were similar and much higher than the MFI given by staining with sera elicited after immunization with OAg-negative GMMA (Fig. 3e). However, the signal given by sera raised against OAg-negative GMMA was low but detectable, even if the binding of these antibodies did not result in killing of OAg-positive bacteria. This signal may be due to the binding of antibodies directed to the lipid A core (as observed in ELISA, see Fig. 3a). When OAg-negative bacteria were stained with the same sera, the differences among the groups reflected those observed in ELISA and SBA (Fig. 3f).

### Identification of the immunodominant protein antigens in GMMA

GMMA surface protein immunoprecipitation (IP) coupled with Mass Spectrometry (MS) was performed with pooled sera from mice immunized with OAg-positive GMMA (Ss Δ*htrB* OAg+ or Ss Δ*msbB* OAg+ GMMA) to detect and identify the immunodominant protein antigens, therefore the antigens on GMMA surface that are mainly recognized by the two sera. GMMA from Ss OAg− were used for the immunoprecipitations and the precipitated proteins were identified and quantified by LC–MS/MS. A total of 15 outer membrane proteins, which are most likely important for the observed serum bactericidal activity, were identified and quantified in the pulled down samples (Table 3). Of these, 7 proteins were pulled down exclusively with sera raised against Ss Δ*htrB* OAg+ GMMA. While among the 8 remaining proteins, 4 antigens were pulled down in greater amounts (at least twofold more) with sera raised against Ss Δ*htrB* OAg+ GMMA compared to sera raised against Ss Δ*msbB* OAg+ GMMA. Taken together, these results confirmed not only that Ss Δ*htrB* OAg+ GMMA elicited higher anti-GMMA proteins antibody titers compared to Ss Δ*msbB* OAg+ GMMA, as also seen by ELISA, SBA and FACS analysis (Fig. 3), but also that the antigens recognized by these antibodies are different. Interestingly, the differences observed in the amount of each antigen pulled down by the two sera do not correlate with the amount of the same antigens in the GMMA used to immunize mice, and despite some of the immunodominant proteins were comparably abundant in both GMMA samples, they were able to induce higher antibody levels when displayed in the context of Ss Δ*htrB* OAg+ GMMA rather than Ss Δ*msbB* OAg+ GMMA.

**Table 3:** Proteins identified through immunoprecipitation of GMMA proteins with sera from groups immunized with OAg-positive GMMA.

Name	Description	MW (kDa)	Fold change in the amount pulled down with sera anti-GMMA Ss Δ*htrB* OAg+ vs. Ss Δ*msbB* OAg+	% of total in Ss Δ*htrB* OAg+ GMMA	% of total in Ss Δ*msbB* OAg+ GMMA
**Outer membrane proteins pulled down with both sera raised against OAg+ GMMA**					
SlyB	Outer membrane lipoprotein	15.6	1.4	0.94	1.35
OmpC	Outer membrane porin protein C	40.5	4.3	31.16	51.36
OmpA	Outer membrane protein A	37.2	15.2	26.87	13.71
BamA	Outer membrane protein assembly factor	90.6	0.7	1.68	1.82
BamB	Outer membrane protein assembly factor	41.9	0.5	0.58	0.55
BamC	Outer membrane protein assembly factor	36.8	0.5	0.85	0.59
BamD	Outer membrane protein assembly factor	27.8	2.1	0.28	0.18
Lpp	Major outer membrane lipoprotein	8.3	6.2	0.67	0.86
**Outer membrane proteins pulled down exclusively with serum raised against Ss ΔhtrB OAg+ GMMA**					
TolC	Outer membrane channel protein	53.7	NA	2.75	1.91
YccZ	Outer membrane lipoprotein group 4 capsule polysaccharide	41.7	NA	0.75	0.16
OmpF	Outer membrane protein F	39.3	NA	1.66	2.00
OmpX	Outer membrane protein X	18.6	NA	0.50	1.99
Pal	Peptidoglycan-associated protein	18.8	NA	0.57	0.49
OmpD	Phosphoporin	39.6	NA	0.76	2.11
BZ172_09410	Uncharacterized protein	22.2	NA	1.09	1.21

GMMA surface immunoprecipitation was performed with pooled sera from mice immunized with OAg-positive GMMA to detect the protein antigens that are mainly recognized by the two sera and therefore to identify the immunodominant protein antigens in both GMMA preparations. OAg-negative GMMA carrying wild-type lipid A were used for the immunoprecipitation experiments and the precipitated proteins were identified and quantified by LC–MS/MS. A total of 15 outer membrane proteins were identified and quantified in the pulled down samples.

NA not applicable.

## Discussion

GMMA have been proposed as an attractive platform for the development of an OAg-based vaccine against *S. sonnei*. *S. sonnei* GMMA have demonstrated to be able to induce high levels of anti-LPS specific IgG both in animal models and in humans^6,14,17,18^. However, the quality and the relative contribution of protein-specific antibodies to the overall immune response induced by GMMA have never been fully elucidated and dissected from the immune response directed to the OAg. Other groups are working on vaccines against *Shigella* which are based on protein antigens^8,22^. Here we tested GMMA differing for the presence and the relative density of OAg chains on their surface, while the length of OAg chains was similar among the OAg-positive GMMA. The two alternative detoxification strategies resulted in different OAg densities on GMMA^23^. Indeed, the *htrB* mutation had a strong impact on the overall OAg amount which was largely decreased compared to the parental strain and the alternative *msbB* double mutant. On the same line, we verified through proteomic analysis that the majority of proteins are similarly represented in the different GMMA samples. Moreover, the proteins detected and their relative abundance were in line with the results obtained for similar OAg-negative GMMA preparations in previous studies^15,24^, further confirming the reproducibility of GMMA production.

When tested in mice, OAg-positive GMMA were able to elicit higher anti-LPS specific antibody titers compared to OAg-negative GMMA, as expected. Anti-LPS total IgG elicited after immunization with OAg-negative GMMA were probably due to the presence of lipid A core on the surface of OAg-negative GMMA as well as in the ELISA plate coating antigen. The group of mice immunized with Ss Δ*msbB* OAg+ GMMA received a dose of OAg which was around eight times higher compared to the group of mice immunized with Ss Δ*htrB* OAg+ GMMA, but the same protein dose. This difference in the OAg dose did not impact on the anti-LPS response because the tested OAg doses were already at the plateau of the LPS-specific humoral response elicited by *S. sonnei* GMMA in mice, as verified in previous dose response studies (data not shown). In order to fully elucidate if OAg density can play a role on the anti-LPS response induced, *ad hoc* studies testing OAg-positive GMMA at lower and not immune-saturating doses, conducted in a dose-response manner, will be necessary and are envisaged together with allometric studies.

On the other hand, despite the same protein dose was used, higher OAg doses corresponded to lower anti-protein responses, suggesting an immuno-interference between OAg and protein antigens. It is known that mice orally inoculated with the same dose of bacteria having reduced LPS synthesis can enhance production of anti-outer membrane proteins antibodies compared to immunization with wild-type bacteria^25^. Moreover, protein immunoprecipitation coupled with mass spectrometry, performed with sera from mice immunized with OAg-positive GMMA, allowed to detect the immunodominant protein antigens in both Ss *ΔhtrB* and SS *ΔmsbB* OAg+ GMMA preparations and suggested that OAg density can have an impact on quantity and quality of the immune response against membrane proteins. These results also confirm that GMMA are not only a delivery system for the *S. sonnei* OAg since they also display additional protein antigens which are strongly immunogenic.

Although an immune correlate of protection against shigellosis has not yet been established, two well characterized assays are available to measure the functionality of the vaccine-induced antibodies against *Shigella* spp: SBA and opsonophagocytic killing (OPK)^10^.

Even though both OAg-positive and OAg-negative GMMA were able to elicit anti-protein antibodies, sera elicited against OAg-positive GMMA were able to kill *S. sonnei* in SBA, whereas sera raised against OAg-negative GMMA did not show any complement-mediated bactericidal activity on OAg-positive *S. sonnei*. Although we cannot rule out the contribution of anti-protein antibodies in OPK, both functional assays follow the same response pattern and have been attributed primarily to antibodies specific for the OAg^26^. In line with this, we observed lack of binding of anti-protein antibodies to OAg-positive bacteria, which is a prerequisite for both complement-mediated or phagocyte-mediated killing. Therefore, both functional assays may not be suitable to assess efficacy for protein-based vaccine candidates. However, by setting up a serum bactericidal assay with an OAg-negative *S. sonnei* strain, we were able to demonstrate for the first time the bactericidal activity of sera that are not targeting the OAg. Indeed, when the bacterial surface proteins are not shielded from antibody binding by the OAg chains, anti-protein antibodies are able to interact with their targets as proven by FACS analysis and to mediate complement deposition and bacterial killing as proven by SBA. Additional studies looking at cellular immune responses would be beneficial to further explore the role of proteins on GMMA immunogenicity.

From this study we concluded that the OAg is indeed a key antigen for functional immunity against *S. sonnei* strains, but antibodies against protein antigens are anyway generated upon immunization with *S. sonnei* GMMA and are functional against *S. sonnei* when bacterial surface is not shielded by OAg chains. This is an important aspect and an added value of GMMA vaccines, since *Shigella* pathogenesis is complex^27^, OAg length is modulated in vivo^28,29^, and antibodies against targets other than OAg might be important in protecting against the spread of the infection in specific organs or time of infection.

## Materials and methods

### Bacterial strains and generation of mutants

*Shigella sonnei* 53G was chosen as parent strains. The list of *Shigella* mutant strains used in this study and their abbreviated identifications are listed in Table 1. Loss of the major virulence plasmid pSS was obtained by selecting a colony with white appearance on congo red agar and genotype confirmed by the absence of the origin of replication and the plasmid encoded gene *wzy*^15^. The null mutation of *msbB1, msbB2*, *htrB*, *tolR*, *wbg*, *virG* was obtained by replacing the gene of interest (“gene”) with an antibiotic resistance cassette or auxotrofic marker, by homologous recombination using lambda red recombeneering system^30^. The *tolR* gene was replaced by kanamycin, the *msbB2*, *htrB* and *wbG* genes were replaced by chloramphenicol, the *msbB1* gene by erythromycin antibiotic cassettes, respectively. The *virG* gene was replaced by the *nadA* and *nadB* genes from *E. coli*. Ss Δ*msbB* OAg+ strain was generated in this study using the same primer sets specific for *tolR*, *msbB1*, and *virG* genes previously reported^12,14,15,20^ in addition to a specific primer set for the replacement of the *msbB2* gene with the chloraphenicol resistance cassette from plasmid pKD3^30^ (Forward primer: taaaatattaatgatgattatggtaggggcattcgcactaaataatgaaaGTGTAGGCTGGAGCTGCTTC, Reverse primer: acaactagtggaaatacctgtactttataatttcaagggtacgggtccgcCATATGAATATCCTCCTTAG).

### GMMA production and characterization

*S. sonnei* GMMA were produced and purified as previously described^14^. Total protein content was estimated by microBCA using bovine serum albumin (BSA) as a reference following the manufacturer’s instructions (Thermo Scientific), total OAg amount by HPAEC-PAD^31^, purity and particle size was detected by HPLC-SEC/MALLS^32^, lipid A structure was determined by MALDI-TOF MS^12^, extracted OAg was characterized by HPLC-SEC and NMR^33^. Behaviour of different GMMA has been also confirmed by MAT using human PBMC^12^. Despite the presence of antibiotic resistance cassettes in the final GMMA-producing strains, antibiotics are not needed during large-scale growth^14^ and possible nucleic acids contaminations in GMMA preparations are monitored and kept below the regulatory acceptance criteria for use in humans.

### Proteomic analysis

For quantitative proteomic analysis, one hundred micrograms of GMMA were TCA precipitated as previously described^34,35^ and the protein pellet was resuspended in 50 mM ammonium bicarbonate containing 0.1% (w/v) RapiGest SF (Waters) and 5 mM DTT and heated at 100 °C for 10 min. Digestions were performed overnight at 37 °C with 2.5 μg trypsin (Promega). Digestions were stopped with 0.1% (v/v) formic acid, desalted using OASIS HLB cartridges (Waters) as described by the manufacturer, dried in a Centrivap Concentrator (Labconco) and resuspended in 50 μL of 3% (v/v) ACN and 0.1% (v/v) formic acid. Peptide mixtures were stored at − 20 °C until further analysis.

For each GMMA sample, three (technical replicates) LC–MS/MS acquisitions were performed as previously described^34,35^. The percentage of each protein in the total sample was calculated according to the corresponding peak area (averaged between the three techincal replicates) and the theoretical molecular weight (MW) using the following formula:

$$\mathrm{\%Protein \, X}=\frac{Avg \, Area \, Protein \, X \, * \, MWProtein \, X}{\sum \left(Avg \, Area \, Protein \, * \, MWProtein\right)}$$

The mass spectrometric raw data were processed with the PEAKS software ver. 8 (Bioinformatics Solutions Inc.) for de novo sequencing, database matching identification and label free quantification, as previously described^35^. Protein identification from MS/MS spectra was performed against *S. sonnei* 53G database (UniProt code UP000194501-4779 ORF entries) combined with common contaminants (human keratins and autoproteolytic fragments of trypsin) with a FDR set at 0.1%. The localization of the quantified proteins was predicted according to PSORTb software ver. 3 (Brinkman Laboratory, https://www.psort.org/psortb/).

### Mouse studies

Animal studies were performed at Toscana Life Science Animal Care Facility under the animal project 479/2017-PR 09/06/2017 approved by the Italian Ministry of Health. Groups of 8 CD1 mice (Female, 5 weeks old) were vaccinated intraperitoneally (i.p.) with 10 µg of GMMA (protein dose based on microBCA quantification) in 200 µL of saline at study day 0 and 28. Approximately 100 µL bleeds (50 µL serum) were collected at day − 1 (pooled sera) and 27 and 42 (individual sera). The number of mice per group was selected based on previous studies with GMMA construct. Indeed, considering a SDLog10 of 0.3, a number of 8 CD1 mice per group has 80% power to detect a fold difference of geomeans equal to 2.8 times between two groups (by using Student's t-test and a = 0.05).

### Ethics

All animal experiments were performed in accordance with good animal practice as defined by the relevant international (Directive of the European Parliament and of the Council on the Protection of Animals Used for Scientific Purposes, Brussels 543/5) and local animal welfare guidelines in Toscana Life Sciences facilities under Italian authorisation.

### Assessment of anti-*S. sonnei* LPS and anti-*S. sonnei* GMMA proteins immune responses in mice

Pre-immune sera and sera collected four weeks after the first and two weeks after the second immunization were analysed by ELISA^14^ for anti-*S. sonnei* LPS total IgG, IgG1, IgG2a, IgG2b, IgG3, IgM content using *S. sonnei* LPS as plate coating antigen (at the concentration of 0.5 µg/mL in Phosphate Buffer Saline, PBS) and for anti-*S. sonnei* GMMA proteins total IgG, IgG1, IgG2a, IgG2b, IgG3, IgM content using Ss OAg- GMMA as plate coating antigen (at the concentration of 1 µg/ml in PBS).

### Assessment of serum bactericidal activity against *S. sonnei*

Single sera collected at day 42 were assayed in SBA based on luminescent readout as previously described^36^ against OAg-positive *S. sonnei* strain (Ss WT OAg+) and OAg-negative *S. sonnei* strain (Ss WT OAg−). Heat inactivated (HI) sera were serially diluted in PBS in the SBA plate (10 µL/well). The starting dilution of each serum in the assay was 1:100 (final dilution) for SBA conducted with *S. sonnei* OAg-positive strain and 1:500 (final dilution) for SBA conducted with *S. sonnei* OAg-negative strain, followed by threefold dilutions steps up to 7 dilution points, plus one control well with no sera added. A 4-parameter non-linear regression was applied to raw luminescence (no normalisation of data was applied) obtained for all the sera dilutions tested for each serum; an arbitrary serum dilution of 10^15^ was assigned to the well containing no sera. Fitting was performed by weighting the data for the inverse of luminescence^2^ and using GraphPad Prism 7 software (GraphPad Software).

Results of the assay are expressed as the IC50, represented by the reciprocal serum dilution that is able to reduce the luminescence signal of 50% compared to the negative control (and thus causes 50% growth inhibition). Titers lower than the minimum measurable assayed were assigned a value of half of the first dilution of sera tested (50 and 250 respectively for SBA assessment of OAg-positive and OAg-negative strains).

### Statistical analysis

Significant differences between ELISA responses and SBA titers were evaluated using the non-parametric two-tailed Mann–Whitney test. The comparison was performed between the two groups immunized with OAg-positive GMMA, the two groups immunized with OAg-negative GMMA and between the groups immunized with GMMA sharing the same lipid A structure.

### FACS analysis

*Shigella sonnei* strains (Ss WT OAg+ and OAg−) were grown overnight at 37 °C in LB supplemented with 20 µg/mL chloramphenicol. Bacteria were then treated as prevoiusly described^35^. They were pelleted and washed with PBS at 8000×*g* for 5 min. Bacteria were then blocked with PBS containing 3% (w/v) Bovine Serum Albumin (BSA) for 15 min and incubated with mouse sera diluted in PBS + 1% (w/v) BSA (1:500) for 1 h. After washes, samples were incubated with Alexa Fluor 647 goat anti-mouse IgG (1:500) (Molecular Probes) for 30 min. Finally, bacteria were fixed with 4% (w/v) formaldehyde for 20 min and flow cytometry analysis was performed using FACS Canto II flow cytometer (BD Biosciences).

### GMMA surface immunoprecipitation

GMMA from Ss OAg− (150 µg) were diluted in 960 µg of PBS and 40 µL of pooled sera were added (either pre-immune sera or sera from mice immunized with the two alternative OAg-positive GMMA carrying penta-acylated lipid A). The reaction mix was incubated for 90 min at 4 °C. After incubation, GMMA lysis was performed by adding 1 mL of solubilization buffer (100 mM Tris, pH 7.8, 300 mM NaCl, 2 mM EDTA, 2% Triton X-100, 0.4% sodium deoxycholate, 0.2% SDS). The mixture was incubated at 37 °C for 60 min followed by 60 min centrifugation at 14,000×*g*.

Protein A/G UltraLink beads (50 µL) were added to the lysates and incubated overnight at 4 °C, following the manufacturer’s protocol. Following overnight incubation, beads were washed twice with solubilization buffer and once with PBS. To elute the immune complexes, 50 μL of Elution Buffer (0.1 M glycine, pH 2.7) were added and incubated for 5 min. pH of eluate was adjusted to physiological values by adding ~ 10 μL of Neutralization Buffer (1 M phosphate pH 9) and beads were separated from the eluate. The samples were analysed by LC–MS/MS as described above.

## Supplementary Information

Supplementary Figures.

Supplementary Tables S1.

Supplementary Table S2.
