# Supplementary material for: Dissecting the contribution of O-Antigen and proteins to the immunogenicity of Shigella sonnei generalized modules for membrane antigens (GMMA)
Source: Sci Rep. 2021 Jan 13;11:906. doi: 10.1038/s41598-020-80421-y (PMC7806729; doi:10.1038/s41598-020-80421-y)
Supplement: Supplementary file 1 — Supplementary Figures. [file 41598_2020_80421_MOESM1_ESM.docx]

**Dissecting the contribution of O-Antigen and proteins to the immunogenicity of *Shigella sonnei* Generalized Modules for Membrane Antigens (GMMA)**

Francesca Mancini^1§^, Gianmarco Gasperini^1§^, Omar Rossi^1^, Maria Grazia Aruta^1^, Maria Michelina Raso^1^, Renzo Alfini^1^, Massimiliano Biagini^2^, Francesca Necchi^1^, Francesca Micoli^1*^

1 GSK Vaccines Institute for Global Health (GVGH), Siena, Italy

2 GSK, Siena, Italy

§ These authors contributed equally

*Corresponding author: Dr Francesca Micoli, GVGH, via Fiorentina 1, 53100, Siena, Italy francesca.x.micoli@gsk.com

**Supplementary Figure S1. Additional GMMA characterization**. HPLC-SEC profiles of GMMA (TSKGEL G4000 PW + TSKGEL G6000 PW columns in series equilibrated in PBS): (upper panel) fluorescence emission profile confirming that GMMA are pure from soluble proteins; (lower panel) traces at 260/280 nm by PDA profile; (b) MALDI-TOF spectra of lipid A extracted from GMMA; (c) Monocite Activation Test (MAT): IL-6 released by human Peripheral Blood Mononuclear Cells (PBMC; commercial from Biopedric) after stimulation with each GMMA. The dashed horizontal black line represents a 10-fold increase over background.

**
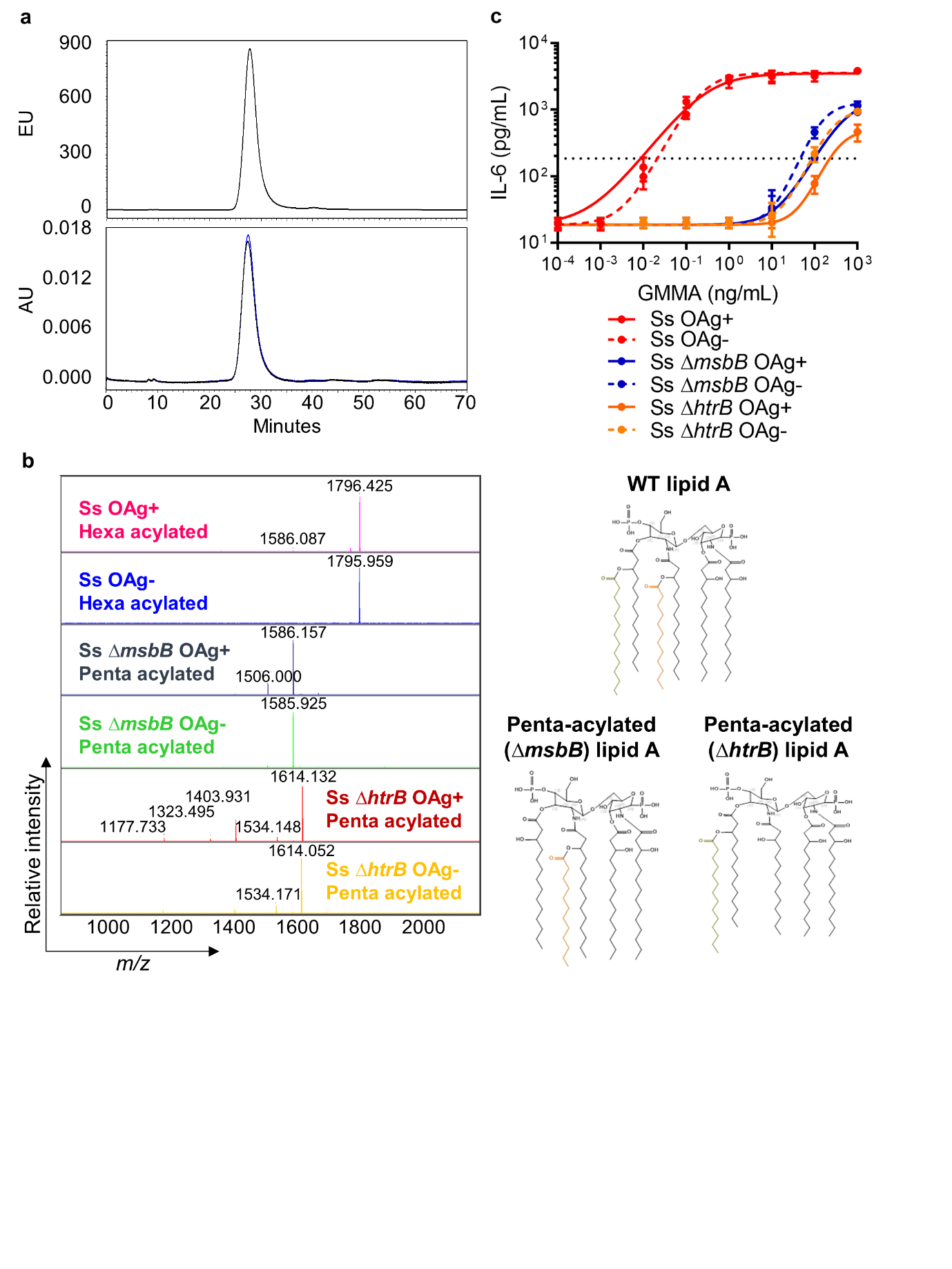
**

**Supplementary Figure S2.** **Immunogenicity study of GMMA in mice: analysis of serum IgM and IgG1/IgG2 content.** Sera have been analysed in ELISA using as coating antigens *S. sonnei* full LPS (a,c) or OAg-negative GMMA (b,d). Results are reported as geometric means (a,b) or as ratio between IgG1 and IgG2 (c,d). Mann-Whitney test was performed between the groups immunized with OAg-positive GMMA, the groups immunized with OAg-negative GMMA and between the groups immunized with GMMA sharing the same lipid A structure (* P<0.05, ** P<0.01, *** P<0.001).

**
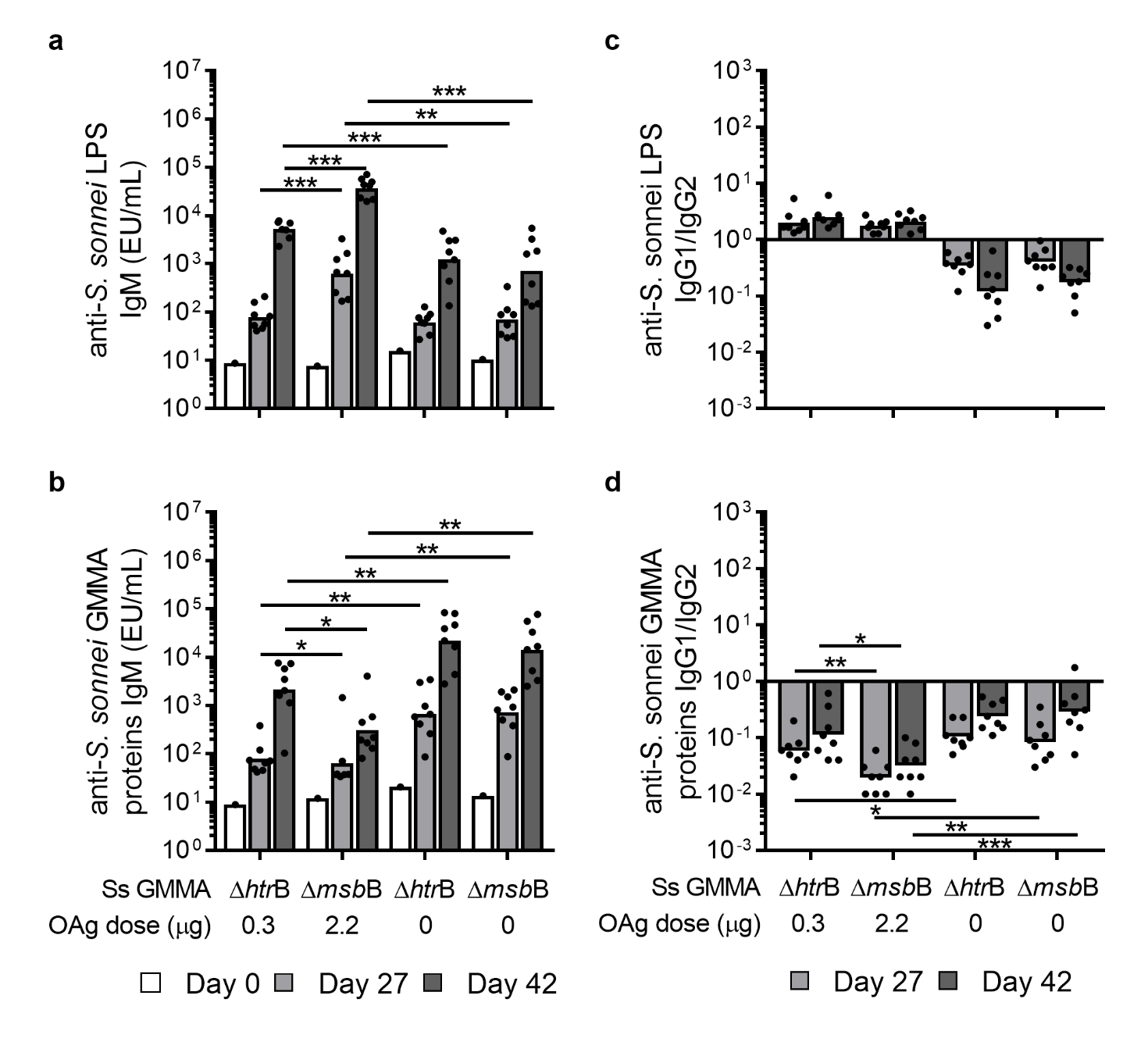
**

**Supplementary Figure S3. Immunogenicity study of GMMA in mice: analysis of serum IgG subclasses.** Sera have been analysed in ELISA using as coating antigens *S. sonnei* full LPS (a-d) or OAg-negative GMMA (e-h). Geometric means and individual values are plotted in the graphs.

**
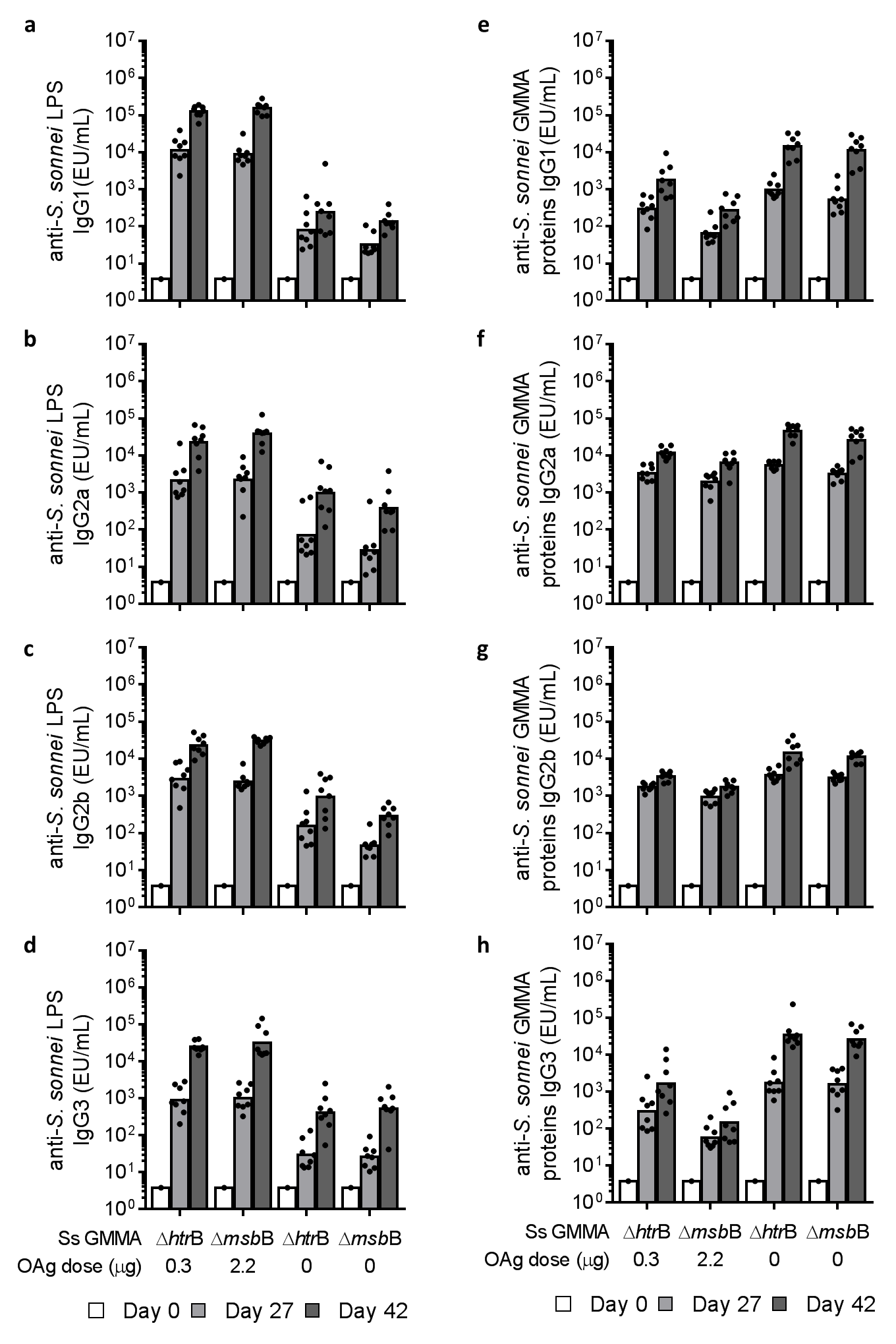
**

**Supplementary Table S1. In depth proteomic analysis of the GMMA protein composition by LC-MS/MS (all data).** The table summarizes all the proteins identified and quantified by LC-MS/MS in the six different GMMA samples analysed.

**Table S2. Proteins identified through proteomic analysis.** Proteomic analysis was performed by LC-MS/MS. The 30 most aboundant proteins in each GMMA were considered and accounted for >95% of the total protein composition of each sample. The localization of the quantified proteins was predicted according to the PSORTb software. C=cytoplasm; OM=outer membrane; P=periplasmic; IM=inner membrane; U=unknown; ND=not detected.
